# Validity and reliability of the Korean version of the Humanism Scale Short Form: A cross‐sectional study

**DOI:** 10.1002/nop2.968

**Published:** 2021-06-23

**Authors:** Hyunjin Lee, Kawoun Seo

**Affiliations:** ^1^ College of Nursing Eulji University Daejeon Korea; ^2^ Department of Nursing Joongbu University Geumsan‐gun Korea

**Keywords:** humanism, instrument, nurses, validity and reliability

## Abstract

**Aim:**

To examine the reliability and validity of a Korean version of the Humanism Scale Short Form.

**Design:**

A cross‐sectional descriptive design.

**Methods:**

This study involved 191 nurses in Korea. Data were collected from 1 May to 30 June 2019. Bilingual nursing professionals translated the scale into Korean, and reverse translation was performed. Validity and reliability were assessed, and the correlation coefficients of the developed scale were compared with those of the Korean version of the Empathy Quotient Scale and Compassion Competence Scale to evaluate concurrent validity.

**Results:**

Exploratory factor analysis with 15 items showed that two factors (human equality and respect for human beings) explained 50.86% of the variance. Cronbach's alpha for the overall scale was 0.88. Thus, the scale has acceptable reliability and validity. Humanism measures can predict a nurse's approach to holistic care and provide fundamental data for developing programs to improve integrated caring capacities.

## 1 INTRODUCTION

Humanism refers to the idea or attitude that human dignity is of the highest value, and that the pursuit of the well‐being and welfare of humankind should transcend racial, national and religious differences (Korea National Institute of Korean Language, 2019). It is based on a view of human beings as unique individuals responsible for each other; this perspective acknowledges people's experiences and emphasizes respect (Kleiman, 2009, p. 56; McCabe, 2007, p. 18). In such a view, attention is focused on individuals as human beings with dignity above all (Park, 2013). In nursing, the approach to care should reflect this (Kang, 2006).

During the course of their work, nurses encounter complex ethical and moral challenges. As nursing knowledge is based on practical experience (Kleiman, 2001; McCamant, 2006; O'Connor, 1993), methods to resolve these challenges can vary depending on the experience of the nurse. Therefore, nurses need to perform caring behaviours with their overall understanding and attitudes informed through the worldview of humanism (Fagermoen, 2006), which integrates key altruistic values.

The values of humanism are often demonstrated in daily nursing care (Paterson & Zderad, 1976, p. 23). Nurses understand their patients through conversation and by displaying empathy, which is indicative of humanistic care. This tendency towards humanistic care has been adequately demonstrated in recent nursing studies on communication and emotional intelligence or empathy, which are necessary for engaging with patients (Ha & Jeon, 2016). Nurses with highly developed nursing skills have been identified as those with a high caring ability, who can empathize with and understand a patient (Jeong & Kim, 2017). In discussions on humanism education in the medical field, there has been an emphasis on the interpersonal factor of communication ability or empathy (Gaufberg & Hodges, 2016). Therefore, for humanistic nursing, it is essential to develop a high caring ability expressed through emotional intelligence or empathy, suitable communication skills, and patient‐centred care (Létourneau et al., 2020).

The Humanism Facet Scale, developed by Nilsson (2015), has emerged as a representative tool for measuring humanism. It is based on existing research on humanism and normative worldviews. Further, it has displayed confirmed reliability and validity in studies conducted with Swedish adults and psychology students from New York University. Initially, this scale consisted of a total of 40 items across five subdomains to measure humanism in general. Subsequently, the scale underwent revisions and, in 2016, the Humanism Scale Short Form was developed, in which 15 items highly correlated with the respective domain scores were extracted. The Humanism Scale Short Form includes items across five subdomains—human nature viewed as good, emotional openness, interpersonal warmth, justice and well‐being, and romantic rationalism—and can be considered suitable for assessing humanistic values and attitudes in nurses. A Korean version of the Humanism Scale Short Form, however, has not yet been tested for reliability and validity.

This study aimed to address this gap by translating Nilsson's Humanism Scale Short Form into Korean and evaluating its suitability for assessing the level of humanism in nurses in clinical practice. The specific study objectives were to: (a) translate the Humanism Scale Short Form into Korean; (b) test the construct validity of the Korean version of the Humanism Scale Short Form; (c) test the reliability of the scale; and (d) test its concurrent validity.

## 2 BACKGROUND

Humanism is a philosophical stance that emphasizes humanity. The word originates from the Latin term, *humanitas*, which means “making people more human” (Veyne, 1993, p. 342). Throughout history, humanist thoughts have found representation both in Eastern (e.g. the Donghak concept of Innaecheon) and Western cultures (e.g. Renaissance period). However, since ancient times, humanism has undergone several changes and has been influenced by other ideologies characteristic of each era. Thus, only the fundamental framework of respect for humanity has been maintained (Doosan Encyclopedia Editorial Staff, 2010).

In the field of human health, a humanistic, person‐centred approach was first proposed in 1940 by American psychologist Carl Rogers (Morgan & Yoder, 2012). This approach was based on the idea that the relationship between counsellor and client should be one of equality. In a similar context, Abdellah (1960) proposed human‐centred nursing because of the need for an individualized approach that incorporated human‐centred values into the profession. This new philosophy differed from the disease‐oriented nursing that prevailed until then; there occurred a shift in focus towards patients' values and needs (Hong, 2018). In order to practice human‐centred nursing, above all, nurses must approach the discipline with humanistic ideals.

The role of humanism in nursing has been reported in many studies on hospice or palliative care, in connection with the extended lifespans seen in recent years. Hopkinson (1999) applied humanistic nursing theory in a study on patients' perceptions of hospice day care. Coward (1991) studied humanism in nurses who cared for patients with breast cancer and found that nurses' humanism affected patients' psychological stability. Although humanism has been criticized for its limited role in promoting science and has been considered objectively indiscernible (Traynor, 2009), it has also been claimed to be a necessary quality among nurses with its emphasis on fostering ideals and practices for improved patient care (Wu & Volker, 2012). Nevertheless, there is no objective tool to measure this competency in nurses in Korea.

## 3 THE STUDY

### 3.1 Design

This was a cross‐sectional descriptive study designed to translate the Humanism Scale Short Form, as revised in 2016, into Korean and test its validity and reliability. Study reporting followed STROBE guidelines (File S1).

### 3.2 Method

#### 3.2.1 Participants

This study was conducted in a general hospital in D city, Korea. The specific participant selection criteria for nurses were as follows: (a) having worked at the general hospital for at least 3 months, (b) patient care as the primary job function, (c) understanding the study's purpose and (d) voluntarily consenting to participate. The required sample size was estimated to be 150 participants, given that a sample size of 5–10 times the final number of items is considered adequate for exploratory factor analysis (EFA; Tab achnick & Fidell, 2001). However, considering possible issues with the survey return rate and response completeness, an online self‐report questionnaire was distributed among 200 nurses. Seven questionnaires were not returned, and two had incomplete responses; thus, a total of 191 questionnaires were available for analysis.

#### 3.2.2 Instruments

##### Humanism scale short form

To assess levels of humanism, the Humanism Scale Short Form was used with permission from the developer. The scale was translated into Korean through a process of translation and back‐translation, and revised based on the results of a test for content validity. The scale consists of 15 items on a seven‐point Likert scale, where 1 = *strongly disagree* and 7 = *strongly agree*. A total score is obtained by summing the items. The higher the total score, the higher the level of humanism. At the time of scale development, the Cronbach's *α*, which is the index for reliability, was 0.93 (Nilsson, 2015).

##### Empathy quotient scale

The Korean version of the Empathy Quotient Scale was used to assess empathy. This scale was originally developed by Baron‐Cohen and Wheelwright (2004), tested for reliability and validity by Lawrence et al., (2004), and translated into Korean by Heo and Lee (2010). This 17‐item scale consists of 10 cognitive empathy items assessing cognitive aspects, three emotional reactivity items assessing emotional aspects, and four social skills items assessing behavioural aspects. All items are rated on a five‐point Likert scale, with a higher total score indicating a higher empathy quotient. In this study, the average score, obtained by dividing the total score by the number of items, was analysed. The Cronbach's *α* was 0.83 in Heo and Lee's (2010) study.

##### Compassion competence scale

Compassion among nurses was assessed using the Compassion Competence Scale developed by Lee and Seomun (2016). This scale consists of a total of 17 items that are rated on a five‐point Likert scale. Total scores range from 17–85 points, and the higher the score, the higher the level of compassion. The Cronbach's *α* value was 0.93 in Lee and Seomun's (2016) study.

#### 3.2.3 Procedure

##### Translation and back‐translation

###### Translation

A double translation method, proposed by Waltz et al. (2010), was used to develop the Korean version of the Humanism Scale Short Form. A nursing professor and a doctor of nursing practice (DNP) with extensive clinical experience, both bilingual in Korean and English, separately and independently translated the Humanism Scale Short Form items, after which they made revisions through comparing the two sets of translations.

###### Back‐translation

A nursing doctoral student, unaware of the contents of the original instrument, whose first language is English and who is also proficient in Korean, translated the Korean items back into English. Then, a nursing professional proficient in both languages compared the back‐translated items against the original instrument to check for any differences. Subsequently, three other nursing professionals examined the Korean wording to test the content validity and finalize items.

##### Pilot test

Before the survey was administered, a pilot test of the Korean version of Humanism Scale Short Form was conducted with 10 university students who were not in the main study sample to ensure readability, comprehensibility and adequacy of the wording. During the process, the survey duration and the reactions of the students while completing the survey were observed. The students were encouraged to give their opinions freely if they found any items unclear or did not understand any aspect of the wording.

##### Test for construct validity

Construct validity was tested using item analysis and EFA. In the item analysis, item means, standard deviation (*SD*), skewness, and kurtosis indices were examined to check the level of bias in the distribution of each item. Additionally, item‐total correlation coefficients were computed to examine whether each item reflected the concept of humanism, and a criterion was established to discard items with a low contribution, that is, a correlation coefficient less than 0.30 (Streiner & Norman, 2011). EFA was performed using principal component analysis, which allows the determination of a stable pattern of consistency coefficients regardless of sample size. A varimax method was used for factor rotation, and the criterion for explained variance was set at 50% or more. Factors with an initial eigenvalue of 1.0 or more were extracted, and items with a coefficient of 0.40 or less were selected (Song, 2011).

##### Reliability analysis

The reliability of the Korean version of the Humanism Scale Short Form was assessed by computing Cronbach's *α*.

##### Test for concurrent validity

Concurrent validity was evaluated by simultaneously administering the Korean version of the Empathy Quotient Scale and the Compassion Competence Scale as these instruments have been reported to be highly correlated with humanism in previous studies (Burks & Kobus, 2012; Gaufberg & Hodges, 2016). Specifically, Pearson's correlation analysis was performed on the total scores of the three instruments to test the extent to which they were correlated.

#### 3.2.4 Analysis

Data were analysed using the SPSS/WIN 22.0 program (IBM Corp.). The participants' demographic characteristics were examined using descriptive statistics. For item analysis, the means, *SD*, skewness, and kurtosis were computed for each item and item‐total correlation coefficients were examined. The goodness of fit of the EFA solution was evaluated using the Kaiser–Meyer–Olkin statistic and Bartlett's test of sphericity. Principal component analysis with a varimax rotation was used to perform EFA. The reliability of the instrument was tested by determining Cronbach's *α* values. Concurrent validity was tested by computing Pearson's correlation coefficients of the Korean version of the Humanism Scale Short Form, the Korean version of the Empathy Quotient Scale, and the Compassion Competence Scale.

#### 3.2.5 Ethics

The study was approved by the institutional review board of the concerned university (JIRB‐2019040801‐01‐190429) and was conducted in accordance with the principles outlined in the Declaration of Helsinki. The selected individuals were first informed of the rationale and purpose of the study, and then an overview of the survey was presented. The survey was administered only to those who provided written informed consent. Participants were assured of anonymity, that the survey data would not be used for purposes other than the research, and that the data would be discarded on completion of the study. The participants were given a small gift for filling out the survey.

## 4 RESULTS

### 4.1 Participants' demographic characteristics

The participants' mean age was 32.12 years (*SD* = *6*.69). Regarding age distribution, 45.5% (*N* = 87) were in their 30 s, the most common age group, followed by those in their 20 s (*N =* 82, 42.9%). Regarding gender, all but two participants were female (*N* = 189, 99%). With respect to religion, most participants (*N =* 123, 64.4%) did not practice any religion, and 12% (*N* = 23) reported pursuing religious activities. Regarding occupational position, most participants (*N* = 173, 90.6%) were staff nurses (Table 1).

**TABLE 1:** Demographic characteristics of participants (*N* = 191)

Characteristics	Categories	*M* (*SD*)	*N* (%)
Gender	Male		2 (1)
	Female		189 (99)
Age (years)	20–29	32.12 (6.69)	82 (42.9)
	30–39		87 (45.5)
	≥ 40		22 (11.5)
Marital status	Married		81 (42.4)
	Unmarried		110 (57.6)
Educational level	College graduate		163 (85.3)
	Master's degree or higher		28 (14.7)
Religion	Yes		68 (35.6)
	No		123 (64.4)
Religious activities	Yes		23 (12)
	No		168 (88)
Position	Staff nurse		173 (90.6)
	Chief nurse		13 (6.8)
	Supervising nurse		5 (2.6)
Department	General ward		85 (44.5)
	Special ward		106 (55.5)
Career (years)	≤2	8.69 (5.60)	23 (12)
	3–9		93 (48.7)
	10–19		66 (34.6)
	≥20		9 (4.7)
Work type	Shift work		130 (68.1)
	Regular work		61 (31.9)
Position satisfaction	Dissatisfied		37 (19.4)
	Neutral		89 (46.6)
	Satisfied		65 (34)
Job satisfaction	Dissatisfied		42 (22)
	Neutral		58 (30.4)
	Satisfied		91 (47.6)
Pay satisfaction	Dissatisfied		109 (57.1)
	Neutral		58 (30.4)
	Satisfied		24 (12.6)

Abbreviations: M, mean; *SD*, standard deviation.

### 4.2 Construct validity analysis

#### 4.2.1 Item analysis

Item analysis and EFA were conducted to evaluate construct validity. The corrected item‐total correlation coefficients of the 15 items computed for the item analysis were in the range of 0.42 to 0.64. No item had a coefficient below the criterion (0.30); therefore, all 15 items underwent EFA. The lowest item mean was 4.51, and the highest was 6.26, while the lowest and highest values for item *SD* were 0.84 and 1.52, respectively. The mean total score was 5.64, and the *SD* was 0.67 (Table 2).

**TABLE 2:** Item analysis and explanatory factor analysis (*N* = 191)

Item	Commonalities	Factor 1	Factor 2	*M* (*SD*)	Skewness	Kurtosis	Corrected item total correlation	*α* If item deleted
Q3	0.63	0.76		5.95 (0.91)	−1.07	1.50	0.62	0.87
Q1	0.59	0.76		6.26 (0.87)	−1.58	3.39	0.42	0.87
Q2	0.56	0.74		5.94 (0.93)	−0.87	0.43	0.55	0.87
Q10	0.57	0.73		6.15 (0.84)	−1.44	3.60	0.58	0.87
Q11	0.53	0.65		6.03 (0.84)	−1.16	2.77	0.62	0.87
Q5	0.50	0.56		5.78 (1.11)	−1.18	1.48	0.63	0.87
Q9	0.34	0.55		6.23 (1.00)	−2.08	6.15	0.45	0.87
Q13	0.45	0.49		5.86 (0.98)	−1.45	3.67	0.60	0.87
Q6	0.26	0.37		5.93 (1.16)	−1.22	1.30	0.43	0.87
Q12	0.53		0.73	4.81 (1.52)	−0.54	−0.23	0.45	0.88
Q15	0.52		0.72	4.51 (1.41)	−0.18	−0.60	0.42	0.88
Q4	0.54		0.71	4.86 (1.35)	−0.46	−0.08	0.56	0.87
Q8	0.55		0.65	5.17 (1.27)	−0.60	−0.08	0.64	0.86
Q7	0.48		0.60	5.54 (1.23)	−0.85	0.39	0.58	0.87
Q14	0.51		0.52	5.71 (0.94)	−0.99	1.17	0.64	0.87
Overall *M* (*SD*): 5.64 (0.67)		6.01 (0.64)	5.10 (0.91)					
Overall Cronbach's *α*: 0.88		0.85	0.80					
Eigenvalue		4.23	3.39					
% variance explained		28.24	22.62					
% cumulative variance explained		28.24	50.86					

Abbreviations: M, mean; *SD*, standard deviation.

#### 4.2.2 EFA

The Kaiser–Meyer–Olkin statistic was 0.88, and the test statistic of Bartlett's test for sphericity was *χ* ^2^ = 1,128.18 (*p* < .001), confirming the goodness of fit of the EFA solution (Lee & Rho, 2015). Factors with an initial eigenvalue of 1 or higher in the principal component analysis were identified, and consequently, two factors were extracted. Item factor loadings ranged between a minimum of 0.37 and maximum of 0.76, and commonalities between a minimum of 0.26 and maximum of 0.63. The eigenvalues were 4.23 and 3.39 for factors 1 and 2, respectively. The explanatory power was 28.24% and 22.62% for factors 1 and 2, respectively, and the total explanatory power was 50.86%. For factor 1, nine items were representative of the equal rights of all human beings in society. Thus, the first factor was named “human equality.” The next six items, in factor 2, consisted of questions that focused on human beings' dignity, respect for individual identity, and respect for others. Therefore, the second factor was named “respect for human beings.” The presence of the two factors in the instrument was confirmed (Table 2), and it was named the “Korean version of the Humanism Scale Short Form.”

### 4.3 Reliability analysis

The test for the internal consistency reliability of the Korean version of the Humanism Scale Short Form showed that the Cronbach's *α* was 0.88 for the entire scale, and 0.85 and 0.80 for factors 1 and 2, respectively (Table 2).

### 4.4 Concurrent validity

The Korean version of the Humanism Scale Short Form was positively correlated with the empathy quotient of the Korean version of the Empathy Quotient Scale (*r* = .42, *p* < .001) and compassion according to the Compassion Competence Scale (*r* = .58, *p* < .001). As the correlation with the reference instruments was between 0.4–0.8, our tool has sufficient concurrent validity as per Lee et al., (2009, p. 572–578).

## 5 DISCUSSION

In the present study, we examined the reliability and validity of a Korean version of the Humanism Scale Short Form, based on the scale developed by Nilsson (2015), further refined in 2016, to objectively assess levels of humanism in nurses in Korea. The test for the reliability of the Korean version showed that the Cronbach's *α* was 0.88, which was higher than the criterion (0.70) proposed by Nunnally and Bernstein (1994). This demonstrates that the instrument was highly reliable. The Cronbach's *α* of the Korean version was found to be similar to the coefficients identified while developing the original instrument—0.83 in a study conducted with US university students, and 0.76 and 0.83 in two studies conducted with Swedish adults (Nilsson, 2015). This finding indicates that the Korean version of the Humanism Scale Short Form is sufficiently reliable for use among nurses in Korea.

The original short scale was created by selecting 15 items loaded to a single factor from a larger scale consisting of 35 items comprising five factors. Although the Korean version of the Humanism Scale Short Form included the original short‐scale items, it seemed more differentiated since two factors were extracted in the EFA. Conducting a between‐study comparison was not possible as factor analysis results regarding construct validity had not been determined when the Humanism Scale Short Form was developed (Nilsson, 2015); however, in this study conducted with nurses, the explanatory power of the items was 50.86%, demonstrating the construct validity of the Korean version. This finding shows that the Korean version of the Humanism Scale Short Form can be used as a foundation for assessing the level of humanism in nurses in more detail.

Correlation coefficients were computed between the Korean version of the Humanism Scale Short Form, the Korean version of the Empathy Quotient Scale, and the Compassion Competence Scale to test for concurrent validity. It was found that humanism was significantly positively correlated with empathy and compassion, which further demonstrated the validity of the Korean version of the Humanism Scale Short Form in assessing levels of humanism in nurses. Particularly, humanism was more strongly correlated with compassion, a competency indicative of professional empathy in nurses (Gaufberg & Hodges, 2016). Accordingly, it appears that the Korean version of the Humanism Scale Short Form is more objective in assessing the level of humanism in nurses, and it provides a fundamental framework to identify the key elements of humanism that are likely to foster compassion.

Based on the study findings, the significance of validity and reliability testing for the Korean version of the Humanism Scale Short Form was as follows. First, the instrument consists of short and easy‐to‐understand items, making it easy to measure the level of humanism in nurses. Second, because the original instrument was translated into Korean using a robust methodology to ensure that the Korean version would accurately reflect it, the current findings can be compared with those based on the Humanism Scale Short Form translated into other languages. Therefore, it would be possible to conduct cross‐cultural studies to compare the level of humanism in nurses in different cultures. Third, even though humanistic elements are stressed in nursing, assessment tools have thus far been unavailable. The Korean version developed in this study is expected to be useful as a measurement instrument as well as a component in interventions for enhancing humanistic competency in nurses by facilitating a more objective assessment of their levels of humanism.

### 5.1 Limitations

Despite these advantages of the instrument, this study had some limitations. First, although the sample size was over 10 times the number of items, the participants were nurses from a single region. Hence, caution is required while generalizing the results, and additional research should be conducted to replicate the findings with a greater number of participants recruited from multiple regions. Second, the instrument was not tested for stability using the test–retest method. Future research is needed to evaluate the stability of the instrument. Third, in this study, we did not conduct a confirmatory factor analysis (CFA). Therefore, CFA is required to further evaluate the tool in further studies.

## 6 CONCLUSIONS

This Korean version of the Humanism Scale Short Form is of considerable significance in that objective data assessing the level of humanism in nurses can be generated. For the clinical application of patient‐centred care, it is essential to study the relationship between nursing and humanism. Accordingly, the Korean version of the Humanism Scale Short Form is expected to be used in future studies to measure the level of humanism, that is, the extent and level of humanistic attitudes, among nurses.

The Korean version of the Humanism Scale Short Form developed in this study is likely to help in assessing humanism in Korean nurses more effectively. Humanism is fundamental for human‐centred care and empathetic understanding in nursing practice. The use of this scale enables a nurse's approach to holistic and integrated care to be more clearly determined in terms of humanism, and can provide fundamental data for the development of programs to improve integrated care.

## CONFLICT OF INTEREST

The authors declare that they have no conflicts of interest.

## Supporting information

File S1

## Data Availability

No data are available in online. All supporting data can be provided upon request to the authors.
